# Associations of anxiety with discomfort and tolerance in Chinese patients undergoing esophagogastroduodenoscopy

**DOI:** 10.1371/journal.pone.0212180

**Published:** 2019-02-19

**Authors:** Man Yang, Ling-Li Lu, Miao Zhao, Jun Liu, Qiu-Lan Li, Qin Li, Peng Xu, Lin Fu, Ling-Min Luo, Jun-Hui He, Wen-Bo Meng, Ping-Guang Lei, Jin-Qiu Yuan

**Affiliations:** 1 Department of Gastroenterology, Songgang People’s Hospital, The Second Hospital Group of Baoan, Shenzhen, Guangdong, China; 2 Special Minimally Invasive Surgery, The First Hospital of Lanzhou University, Lanzhou, China; 3 Scientific Research Centre, The Seventh Affiliated Hospital, Sun Yat-sen University, Shenzhen, Guangdong, China; 4 Division of Epidemiology, JC School of Public Health and Primary Care, The Chinese University of Hong Kong, Hong Kong; University Hospital Llandough, UNITED KINGDOM

## Abstract

**Objectives:**

To evaluate the associations of pre-endoscopy anxiety with discomfort and tolerance in patients undergoing unsedated esophagogastroduodenoscopy (EGD).

**Methods:**

This is a hospital-based cohort study of 348 patients undergoing routine, non-advanced EGD without sedation. The primary outcomes were discomfort and tolerance. The anxiety before endoscopy was evaluated with a 10-point visual analogue scale (VAS). The associations of pre-endoscopy anxiety with the outcomes were evaluated with logistic regression adjusting for potential confounders like age, sex, and body mass index.

**Results:**

Seventy patients reported severe discomfort and 56 patients reported poor tolerance after endoscopy. The risk of severe discomfort increased with pre-endoscopy anxiety and reached a platform around 7–10 points. Compared with the participants with low pre-endoscopy anxiety, those with moderate (adjusted odds ratio [OR] 2.70, 95% confidence interval [CI] 1.17 to 6.22) and high level of anxiety (adjusted OR 6.87, 95% CI 2.16 to 21.79) were associated with a gradual increase in the risk of severe discomfort (P-trend < 0.001). The association between pre-endoscopy anxiety and tolerance was linear, with an adjusted OR of 1.67(95% CI 1.33 to 2.08) for a 1-score increase in pre-endoscopy anxiety VAS. The associations were not modified by age, sex, pharyngitis, duration of endoscopy, and diameter of the endoscope.

**Conclusions:**

Pre-endoscopy anxiety was an independent predictor of severe discomfort and poor tolerance in Chinese patients undergoing unsedated EGD. Our findings suggested the importance of the management of anxiety to reduce adverse endoscopic experience and taking high level of anxiety as an indication for sedation.

## Introduction

Esophagogastroduodenoscopy (EGD) is the gold standard test for the investigation of upper gastrointestinal symptoms, allowing a direct view of mucosal surfaces, photography, biopsy, and therapeutic intervention[1]. EGD is widely accepted. Approximately 6.9 million procedures were performed in the U.S. in 2009[2]. EGD is an invasive and unpleasant procedure which may lead to gag reflex, panic, fear, abdominal fullness and pain[3]. In a survey of 509 patients attending for routine diagnostic gastroscopy, 39 subjects (8%) failed to complete the initial unsedated endoscopy due to poor tolerance, 51 (9%) of those who completed endoscopy experienced severe discomfort during the procedure[4]. The discomfort and tolerance can be effectively improved by sedation, but sedation may lead to adverse events such as cardiorespiratory arrest[5], increase the complexity and duration of endoscopy, and increase medical cost. Identifying risk factors for severe discomfort and poor tolerance is important for clinical practice as it enables individualized use of sedation and, when the factors are modifiable, provides methods to reduce the risk.

A number of factors, such as age[4, 6, 7], pharyngeal sensitivity[7], chronic use of psychotropic drugs or alcohol[8], and diameter of EGD[4] have been shown to be associated with discomfort and tolerance in patients undergoing EGD. As a modifiable factor, anxiety before endoscopy has been investigated in some studies but the evidence remains unclear[4, 6–12]. First, previous studies have showed mixed findings demonstrating either a harmful[4, 7–9], or null effect[6, 10–12] of anxiety on comfort or tolerance. Second, the associations of pre-endoscopy anxiety with discomfort and tolerance may not be linear but no previous studies have investigated the non-linear association. Third, the associations were likely to be confounded by pharyngeal sensitivity, obesity, duration of endoscopy, and endoscopists, but most previous studies did not adequately control these factors[4, 6–12]. Last, the discomfort and tolerance to EGD and acceptance to sedation vary considerably among patients in different countries[13]. Most previous studies were carried out in Europe and North America[4, 6–9, 12], while evidence from the Chinese population has been lacking. Managing anxiety is important for both sedated and unsedated EGD. For unsedated procedures, managing anxiety may reduce the potential needs for sedation; For procedures performed with sedation, it may reduce the doses of sedation, which increases the risk of adverse events and the cost. The aim of the present study was to investigate the associations of pre-endoscopy anxiety with discomfort and tolerance in Chinese patients undergoing unsedated EGD.

## Materials and methods

### Design, study setting, and participants

This is a prospective hospital-based cohort study. We consecutively recruited 348 inpatients or outpatients undergoing EGD from an upper second-class hospital in Shenzhen, China from May to June 2017. The inclusion criteria were as follows: 1) aged 18 years or over; 2) scheduled to undergo routine, diagnostic non-advanced EGD, for any reasons; 3) unsedated; 4) undergoing EGD for the first time. We excluded patients with EGD contraindications including pregnancy, esophageal stenosis, upper gastrointestinal tract anomalies, and a history of upper gastrointestinal surgery. Before recommending an EGD to a patient in our hospital, the doctor needs to ask whether the patient has these contraindications. The judgment for esophageal stenosis and upper GI tract anomalies was based on self-reporting. We cannot rule out undiagnosed esophageal stenosis or upper GI tract anomalies, however, we did not find such cases among the included participants after EGD exam. Written informed consents were obtained from all participants. This study was approved by the ethics committee of Songgang People’s Hospital (SGPHE201704G).

There were two comparisons in our primary analyses (moderate anxiety group vs. low anxiety group, high anxiety group vs. low anxiety group). Based on the estimated effects from previous studies[4, 8], we expected the intolerance rates are 9.3%, 40% and 55% for the low, moderate and high anxiety group, respectively. Using a significance level (2-sided) of 0.05 and a power of 0.8, the total number of participants required is 109 (72 for the comparison _moderate vs low_ and 36 for comparison _high vs low_).

### Endoscopic procedure

To avoid fasting for long period of time for patients, over 95% diagnostic EGD procedures are scheduled in the morning working period (8:00 am to 12:30 am) in our hospital. Endoscopy nurses introduced the EGD procedure, benefits, and potential harms to all participants as a standard procedure before signing the informed consent form for endoscopy. All patients received lidocaine hydrochloride mucilage (12 mg per application) in a standardized fashion about 10 to 20 minutes before the endoscopy. Patients who required sedation were excluded. Five certified endoscopists with at least three years of endoscopy experience performed all procedures. We assigned patients to endoscopists randomly. Patients had no information on the background of the endoscopists. Patients were examined using one of the endoscopes in our center including GIF-H260Z, GIF-Q260J, GIF-Q260, GIF-XQ260 instruments (Olympus, KeyMed, Southend-on-Sea, UK) in the left lateral position. The procedures were performed in a standardized way. An examination was considered technically adequate if all anatomic segments of the upper GI tract (esophagus, stomach, bulb and second portion of duodenum, fundus of the stomach) were adequately viewed.

### Assessment of pre-endoscopy anxiety

We evaluated patients’ level of anxiety in the waiting room before undergoing endoscopy. Participants were asked “what’s your current level of anxiety about the endoscopy?”. The participants were asked to rate their anxiety by a visual analogue scale (VAS) as previous studies [7, 8, 14] (0 point: no anxiety, 10 points: extreme anxiety). We categorized 0 ≤ VAS ≤ 3 as low level, 4 ≤ VAS ≤ 6 as moderate, and 7 ≤ VAS ≤ 10 as high level of anxiety for data analyses.

### Covariates

We selected covariates that may influence patients’ comfort and tolerance based on a literature review [3, 4, 6–8, 11, 15–17] and a discussion with the endoscopists in our center. Data were collected with a structured questionnaire. The covariates included sociodemographic characteristics (age, sex, weigh, height, education, and family income), lifestyle behaviors (smoking, alcohol drinking), current or recent use of psychotropic drugs (antidepressant, antianxiety drug, antimanic drug, antipsychotic drug, tranquillizers, and others), self-reported snore, current diagnose with pharyngitis, and self-evaluated tolerance for uncomfortable feelings such as pain and nausea (evaluated with a 10-point VAS) [7, 8, 14]. Endoscopy nurses with at least 5 years of working experience evaluated the pharyngeal sensitivity by the method described by Moulton et al[18]. Modified mallampati classification was used to evaluate the view of oropharyngeal[19]. The duration of procedure and diameter of endoscope were recorded after the procedures.

### Outcomes

Endoscopy nurses evaluated the study outcomes with a questionnaire about 10 to 20 minutes after endoscopy when the patients were waiting for the EGD results. The primary outcomes were discomfort (determined by asking ‘what was your level of discomfort during the procedure?’) and tolerance (‘how hard did you feel to tolerate the discomfort during the procedure?’). The secondary outcomes included panic and fear (‘what was your level of panic and fear during the procedure?’) and the willingness to repeat unsedated endoscopy in the future. Discomfort, tolerance, and panic/fear were evaluated with a 10-point VAS as used by previous studies[7, 8, 14], and were dichotomized based on a cut-off at 7 point (7 ≤ VAS score ≤ 10: severe discomfort / poor tolerance / intense panic and fear). The willingness to repeat unsedated endoscopy in the future was assessed by answering ‘yes’ or ‘no’.

We evaluated the content validity of the questionnaires by discussing with the five gastroenterologists in our center. Most of the questions were concise and straightforward and have been validated in previous study[12].

### Data analyses

We carried out descriptive analysis and reported means and standard deviations (SD) for continuous variables and percentages for categorical variables. To explore the shape of the association between pre-endoscopy anxiety and study outcomes, we used general additive logistic regression model taking pre-endoscopy anxiety as a smoothed term[20]. The choice of the degrees of freedom was determined by comparing the Akaike Information Criterion and residual deviance of different models[21]. Because no single parameter values for the exposure were returned directly from general additive model, we additionally evaluated the odds ratios (ORs) with regular logistic regression.

Multivariate regression analyses were adopted to adjust for established and potential confounding factors. The basic model adjusted for baseline age (continuous), sex (men or women), and body mass index (continuous). The fully-adjusted model additionally adjusted for current smoking (yes or no), current alcohol drinking (yes or no), education (secondary school or lower, high school or higher), self-reported pharyngitis (yes or no), self-evaluated tolerance to unpleasant feelings (continuous), pharyngeal sensitivity (normal or attenuated), modified Mallampati classification of oropharyngeal view (continuous), duration of endoscopy (continuous), and diameter of endoscope (9.0–9.2 mm or 9.8–9.9 mm). Because the rate of psychotropic drug use was very low (4.6%, n = 16), and the rate of individual drug use was even lower, their contributions to the regression model were tiny. We therefore did not include the use of psychotropic drugs in the regression analysis. The primary analyses were based on complete case analysis. To investigate the potential influence of missing covariate data, we used multiple imputation to generate missing data and reanalyzed the study. We also re-fitted the unadjusted model and basic adjustment model by restricting the participants in those participants with complete data.

We undertook subgroup analyses by age, sex, self-reported pharyngitis, duration of endoscopy, and diameter of endoscope. The interaction effects were tested by including an interaction term in the regression model. We conducted a series of sensitivity analyses to check the robustness of the primary results: 1) additionally adjusting for family income; 2) additionally adjusting for snore; 3) additionally adjusting for endoscopists; and 4) considered VAS ≥ 5 as the definition of severe discomfort, poor tolerance, and intense panic and fear. Two-sided P<0.05 was considered statistically significant for all analyses. Analyses were performed using R software version 3.4.1 (R Development Core Team, 2017).

## Results

### Baseline characteristics

Table 1 presents the baseline characteristics of participants by the level of pre-endoscopy anxiety. The median age of participants was 35 years and 62.1% of the participants were men. The mean pre-endoscopy anxiety VAS score was 4 points (SD = 2). A total of 38 patients were classified into high anxiety group based on the cut-off at ≥ 7 VAS score. The participants with high pre-endoscopy anxiety were likely to be older, with lower self-evaluated tolerance to unpleasant feelings. All participants completed post-endoscopy outcome assessment. Two patients failed to finish the procedure due to severe discomfort. They were considered as cases with severe discomfort, poor tolerance, and severe panic and fear in our analysis; They also expressed unwillingness to repeat unsedated endoscopy in the future. These two patients did not receive sedation to accomplish the procedures so they were still eligible for analysis.

**Table 1:** Characteristics of participants.

	Pre-endoscopy anxiety level			
	Low (0 ≤ VAS ≤ 3) N = 166	Moderate (4 ≤ VAS ≤ 6) N = 144	High (7 ≤ VAS ≤ 10) N = 38	P-value*
Mean (SD) age, years	37.0(12.0)	33.0(9.0)	33.0(6.0)	0.006
Female, n(%)	60(36.1)	62(43.1)	12(31.6)	0.30
Mean (SD) BMI, kg/m^2^	21.9(2.7)	22.3(3.6)	22.4(3.0)	0.58
Current smoker, n(%)	38(24.4)	44(31.0)	12(31.6)	0.39
Current alcohol drinker, n(%)	28(17.9)	36(26.9)	12(31.6)	0.09
Self-reported pharyngitis, n(%)	30(18.3)	34(23.9)	12(33.3)	0.12
Psychotropic medicine, n(%)	6(3.7)	8(5.7)	2(5.3)	0.70
Education				
Illiteracy, n(%)	4(2.5)	0(0.0)	0(0.0)	0.42
Primary school, n(%)	10(6.2)	12(8.3)	4(10.5)	
Secondary school, n(%)	80(49.4)	76(52.8)	20(52.6)	
High school, n(%)	58(35.8)	48(33.3)	14(36.8)	
University or above, n(%)	10(6.2)	8(5.6)	0(0.0)	
Mean (SD) self-evaluated tolerance	5.3(1.4)	4.8(1.0)	4.9(2.8)	0.006
Gag reflex				
Normal, n(%)	154(93.9)	134(94.4)	38(100.0)	0.30
Attenuated, n(%)	10(6.1)	8(5.6)	0(0.0)	
Absent, n(%)	0(0.0)	0(0.0)	0(0.0)	
Mallampati classification				
Class I, n(%)	78(47.0)	66(47.1)	30(78.9)	0.006
Class II, n(%)	66(39.8)	52(37.1)	2(5.3)	
Class III, n(%)	14(8.4)	14(10.0)	4(10.5)	
Class IV, n(%)	8(4.8)	8(5.7)	2(5.3)	
Mean (SD) of endoscopy procedure time, minutes	5.1(2.2)	5.1(2.6)	5.0(2.9)	0.94
Diameter of endoscope				
9.0–9.2 mm	138(87.3)	124(87.3)	22(61.1)	<0.001
9.8–9.9 mm	20(12.7)	18(12.7)	14(38.9)	

*The difference between groups were tested by analysis of variance for continuous variables and Chi-square test for categorical variables

VAS: visual analogue scale (0–10 points). SD: standard deviation. BMI: body mass index

### Discomfort

**Fig 1:**
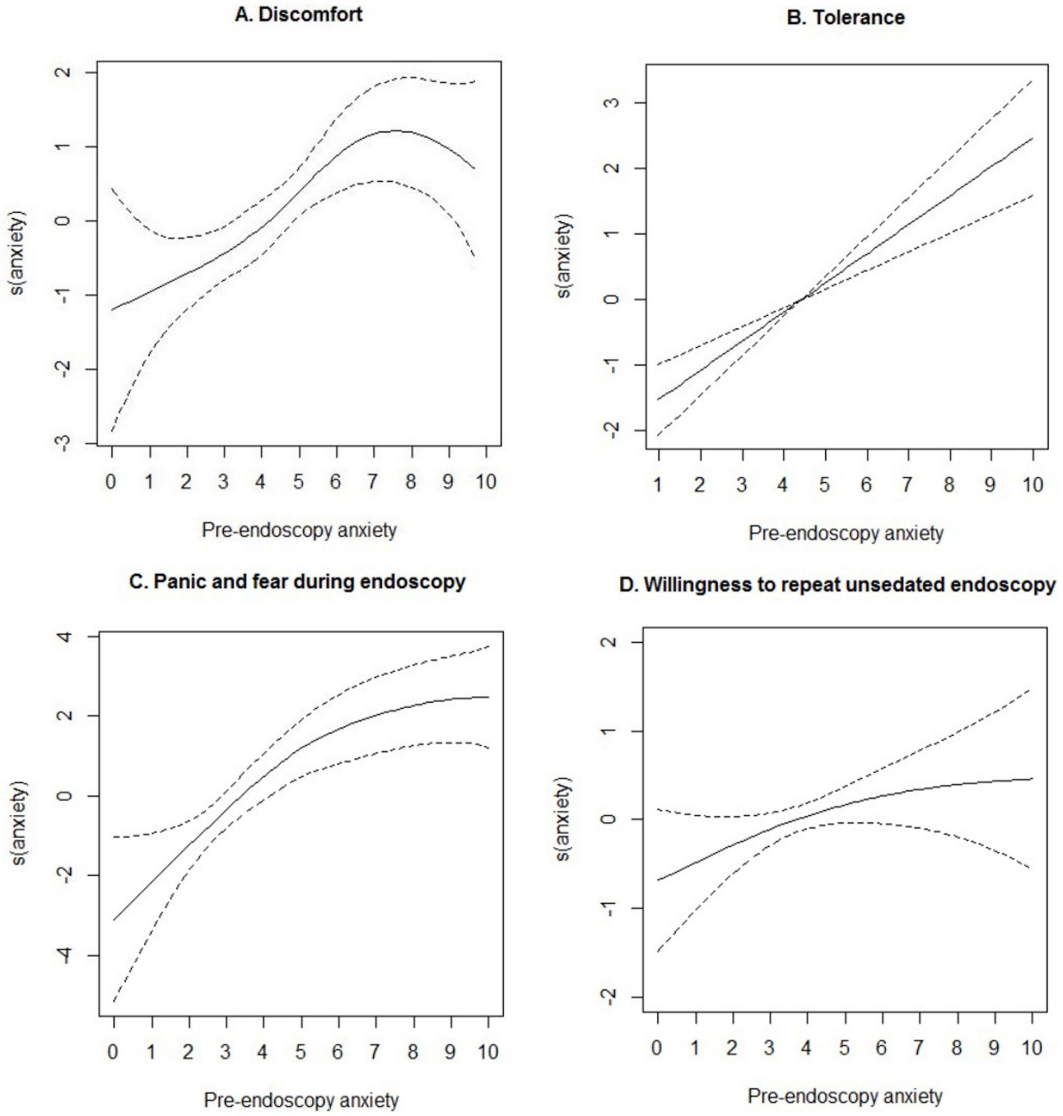
Non-linear associations between pre-endoscopy anxiety with patient-related outcomes. The results were based on general additive model taking pre-endoscopy anxiety score as the smoothing term. The figures showed the smooth component (pre-endoscopy anxiety) of fitted general additive models in terms of discomfort (Panel A), tolerance (Panel B), panic and fear during endoscopy (Panel C), and the willingness to repeat unsedated endoscopy (Panel D). A larger s (anxiety) value indicated greater discomfort, lower tolerance, greater panic and fear during endoscopy, and lower willingness to repeat unsedated endoscopy.

A total of 348 participants with 70 cases of severe discomfort contributed to the analyses. Fig 1A presents the nonlinear association between pre-endoscopy anxiety and discomfort. The association ascended and reached a platform around 7–10 points. Results from regular logistic model suggested that the crude OR for 1-score increase in pre-endoscopy anxiety VAS was 1.33 (95%CI, 1.17 to 1.52)(Table 2). The association did not change after the adjustment for potential confounders (adjusted OR 1.32, 95%CI 1.09 to1.61). Compared with the participants with low pre-endoscopy anxiety, those with moderate (adjusted OR 2.70, 95%CI 1.17 to 6.22) and high (adjusted OR 6.87, 95%CI 2.16 to 21.79) anxiety were associated with a gradual increase in the risk of severe discomfort (P-trend < 0.001). The results were similar in the additional analyses using the multiple imputation dataset or limiting the participants among those with complete covariate information *(*S1 and S2 Tables *in the Supporting Information file*).

**Table 2:** Associations of pre-endoscopy anxiety with discomfort and tolerance.

	OR [95%CI]		
	Unadjusted model	Basic model ^†^	Fully adjusted model ^‡^
**Discomfort**			
No. of participants	348	322	264
No. of events	70	68	56
OR for 1-score increase in pre-endoscopy anxiety VAS	1.33[1.17, 1.52]***	1.31[1.15, 1.51]***	1.32[1.09, 1.61]**
OR by pre-endoscopy anxiety categories			
*Low (0 ≤ VAS* ≤ *3)*	1.00(referent)	1.00(referent)	1.00(referent)
*Moderate (4 ≤ VAS ≤ 6)*	2.33[1.27, 4.27]**	2.37[1.26, 4.48]**	2.70[1.17, 6.22]*
*High (7 ≤ VAS ≤ 10)*	5.38[2.43, 11.92]***	5.12[2.26, 11.60]***	6.87[2.16, 21.79]**
P-trend	<0.001	<0.001	0.001
**Tolerance**			
No. of participants	348	322	264
No. of events	56	54	46
OR for 1-score increase in pre-endoscopy anxiety VAS	1.51[1.30, 1.76]***	1.50[1.29, 1.75]***	1.67[1.33, 2.08]***
OR by pre-endoscopy anxiety categories			
*Low (0 ≤ VAS* ≤ *3)*	1.00(referent)	1.00(referent)	1.00(referent)
*Moderate (4 ≤ VAS ≤ 6)*	3.88[1.81, 8.31]***	3.67[1.66, 8.12]***	4.58[1.53, 13.70]**
*High (7 ≤ VAS ≤ 10)*	14.22[5.77, 35.05]***	13.47[5.33, 34.04]***	30.78[7.90, 119.85]***
P-trend	<0.001	<0.001	<0.001

^†^ Basic model: adjusted for age, sex, and body mass index.

^**‡**^ Fully adjusted model: additionally adjusted for smoking, alcohol drinking, education, self-reported pharyngitis, self-evaluated tolerance to unpleasant feelings, pharyngeal sensitivity, modified Mallampati classification of oropharyngeal view, duration of endoscopy, and diameter of endoscope.

* 0.01 ≤ P < 0.05,

** 0.001 ≤ P < 0.01,

*** P < 0.001

OR: odds ratio. CI: confidence interval. VAS: visual analogue scale (0–10 points)

### Tolerance

A total of 348 participants with 56 cases of poor tolerance contributed to the analyses for tolerance. The relationship between pre-endoscopy anxiety and risk of poor tolerance was linear (Fig 1B), with an adjusted OR of 1.67(95%CI, 1.33 to 2.08) for 1-score increase in pre-endoscopy anxiety (Table 2). Compared with the participants with low pre-endoscopy anxiety, those with moderate (adjusted OR 4.58, 95%CI 1.53 to 13.70) and high (adjusted OR 30.78, 95%CI 7.90 to 119.85) anxiety were associated with a significant increase in the risk of poor tolerance.

### Panic and fear during endoscopy

Forty patients (11.5%) reported severe panic and fear during endoscopy. The association between pre-endoscopy anxiety and risk of severe panic/fear was linear (Fig 1C). The adjusted OR for 1-score increase in pre-endoscopy anxiety VAS score was 1.80 (95%CI 1.39 to 2.32) (Table 3). Moderate (adjusted OR 16.59, 95%CI 3.23 to 85.17) and high (adjusted OR 88.66, 95%CI 12.32 to 637.94) pre-endoscopy anxiety were associated with severe panic and fear during endoscopy when compared with those with low level of anxiety.

**Table 3:** Associations of pre-endoscopy anxiety with panic/fear during endoscopy and the willingness to repeat unsedated endoscopy.

	OR [95%CI]		
	Unadjusted model	Basic model ^†^	Fully adjusted model ^‡^
**Panic and fear during endoscopy**	348	322	264
No. of participants	40	38	34
No. of events			
OR for 1-score increase in pre-endoscopy anxiety VAS	1.58[1.33, 1.86]***	1.57[1.31, 1.87]***	1.80[1.39, 2.32]***
OR by pre-endoscopy anxiety categories			
*Low (0 ≤ VAS* ≤ *3)*	1.00(referent)	1.00(referent)	1.00(referent)
*Moderate (4 ≤ VAS ≤ 6)*	18.60[4.33, 79.92]***	14.69[3.38, 63.82]***	16.59[3.23, 85.17]**
*High (7 ≤ VAS ≤ 10)*	38.31[8.11, 181.02]***	34.62[7.24, 165.60]***	88.66[12.32, 637.94]***
P-trend	<0.001	<0.001	<0.001
**Willingness to repeat unsedated endoscopy**			
No. of participants	342	316	260
No. of events	76	66	60
OR for 1-score increase in pre-endoscopy anxiety VAS	1.14[1.00, 1.29]*	1.12[0.98, 1.28]	1.18[0.99, 1.39]
OR by pre-endoscopy anxiety categories			
*Low (0 ≤ VAS* ≤ *3)*	1.00(referent)	1.00(referent)	1.00(referent)
*Moderate (4 ≤ VAS ≤ 6)*	1.34[0.78, 2.32]	1.29[0.71, 2.34]	1.61[0.76, 3.39]
*High (7 ≤ VAS ≤ 10)*	1.61[0.71, 3.68]	1.75[0.75, 4.11]	1.70[0.57, 5.04]
P-trend	0.21	0.21	0.23

^†^ Basic model: adjusted for age, sex, and body mass index.

^**‡**^ Fully adjusted model: additionally adjusted for smoking, alcohol drinking, education, self-reported pharyngitis, self-evaluated tolerance to unpleasant feelings, pharyngeal sensitivity, modified Mallampati classification of oropharyngeal view, duration of endoscopy, and diameter of endoscope.

* 0.01 ≤ P < 0.05,

** 0.001 ≤ P < 0.01,

*** P < 0.001

OR: odds ratio. CI: confidence interval.VAS: visual analogue scale (0–10 points)

### Willingness to repeat unsedated endoscopy

The unadjusted model suggested that pre-endoscopy anxiety was associated with a higher rate of willingness to repeat unsedated endoscopy, but the association was weak and there was insufficient evidence of association after adjustment for confounders.

### Subgroup analyses and sensitivity analyses

S3 and S4 Tables *in the Supporting Information file* present subgroup analyses. The associations of pre-endoscopy anxiety with discomfort, tolerance and panic/fear were not modified by age, sex, pharyngitis, duration of endoscopy, and diameter of endoscope. The duration of endoscopy (P = 0.05) and diameter of endoscope (P = 0.006) showed interaction effects with the association between pre-endoscopy anxiety and the willingness to repeat unsedated endoscopy. Pre-endoscopy anxiety was associated with a significantly lower rate of willingness to repeat unsedated endoscopy if the duration of endoscopy was ≥ 5 minutes (OR 1.40, 95%CI 1.08 to 1.81) or the diameter of endoscope was 9.8–9.9 mm (OR 1.68, 95%CI 1.18 to 2.39).

Sensitivity analyses by additionally adjusting for family income, snore, and endoscopists generally show no major influence on the primary results (S5 Table *in the Supporting Information file*. The associations did not change taking VAS ≥ 5 as the definition of severe discomfort, poor tolerance, and intense panic and fear.

## Discussion

This hospital-based cohort indicated that the anxiety before endoscopy was independently associated with discomfort, tolerance, panic and fear during endoscopy in Chinese patients undergoing standard diagnostic EGD without sedation. The level of discomfort increased with pre-endoscopy anxiety and reached a platform around 7–10 points. The associations between pre-endoscopy anxiety with tolerance and panic/fear during endoscopy were linear. The association between pre-endoscopy anxiety with the willingness to repeat unsedated endoscopy was modified by the duration of endoscopy and diameter of endoscope. Pre-endoscopy anxiety was associated with a significantly lower rate of willingness to repeat unsedated endoscopy if the duration of endoscopy was ≥ 5 minutes or a relatively larger diameter endoscope (9.8–9.9 mm) was used.

Our findings were in agreement with a number of previous studies[4, 7, 8]. A cohort study of 509 participants suggested that high level of anxiety were related to poor tolerance in patients undergoing diagnostic EGD[7].A study of 508 patients attending for routine diagnostic gastroscopy suggested high apprehension about examination was associated with significantly high discomfort (OR 2.72, 95%CI 1.79–4.12) and preference for sedation during any future endoscopy (OR 2.25, 95%CI 1.44–3.50). However, this study did not find a significant association between apprehension with tolerance (P = 0.51) [4]. A study of 148 participants suggested that nervousness (*P* = 0.02) before endoscopy was significantly associated with adverse endoscopic experience (defined as a score of ≥ 5 on the postprocedure overall level of satisfaction (evaluated with a 10-points scale) or the willingness to repeat endoscopy) in patients undergoing non-advanced endoscopic procedures with conscious sedation[8]. However, in the study by Abraham et al[6], high anxiety (OR 0.49, 95% CI 0.30 to 0.81) was associated with a less comfortable examination in the univariate analyses but there was no association after the adjustment for confounders. A study of 300 Iranians showed that self-reported EGD tolerance during and after an EGD procedure did not correlate with fear or anxiety about the procedure[10]. Possible explanations for these inconsistent results included 1) different adjustments for confounding factors, 2) different cultural and acceptance of unsedated EGD in patients from different countries, and 3) different patient characteristics such as age, sex, and prior endoscopy experience.

This is the first study evaluating the non-linear relationship between pre-endoscopy anxiety with adverse endoscopic experiences in Chinese patients undergoing unsedated EGD. The strengths of this study included the prospective hospital-based study design, subgroup analyses by various factors, and stable sensitivity analyses.

Our study has limitations. First, despite our best efforts to adjust for established and potential risk factors, residual confounding by other unmeasured or unknown factors remains possible. Second, we were unable to evaluate the effect by risk ratio, which is easier for interpretation, since the data failed to converge to the maximum likelihood estimate even with the expectation–maximization algorithm[22].ORs in this study could not be used to directly estimate risk ratios as the outcome rates are high. Third, we were unable to develop a prediction model due to insufficient sample size. Nevertheless, a study to develop such model specific for Chinese patients is ongoing in our center. Last, we did not specify the reasons for EGD, which was likely to influence the pre-endoscopy anxiety level and confound the association between pre-endoscopy anxiety and panic/fear during endoscopy. Anxiety is the exposure in this study and we have specified that the anxiety we evaluated was about the procedure. Though we cannot rule out the confounding effect for panic/fear during endoscopy, the conclusion was unlikely to be altered due to the large effect size, clear dose-response relationship, and the consistency with other endpoints which were unlikely to be influenced by reasons of EGD, such as discomfort and tolerance.

Our findings have important implications on clinical practice and future research. First, this study suggested that high level of pre-endoscopy anxiety may be considered as an indication for sedation in Chinese population. A number of studies have established the role of anxiety as a predictor of selecting patient undertaking EGD with sedation in Spaniards[7], Americans[8], and Britons[4]. Such method may also be applied for Chinese patients although further research is required to develop a valid model adapting to this population. In addition, our study supports appropriate management of anxiety before endoscopy to alleviate discomfort and improve tolerance. Potentially effective interventions include oral midazolam[23], music[24], and written educational material[25]. Further research is needed to evaluate the effectiveness of these interventions in Chinese patients.

In conclusion, this study suggested that the anxiety level before EGD was independently associated with discomfort, tolerance, and panic/fear during endoscopy in Chinese patients undergoing standard unsedated EGD. When the duration of endoscopy procedure was ≥ 5 minutes or a relatively larger diameter endoscope (9.8–9.9 mm) was used, pre-endoscopy anxiety was also associated with a significantly lower rate of willingness to repeat unsedated endoscopy in the future.

## Supporting information

S1 Table Additional analyses of the associations of pre-endoscopy anxiety with study outcomes using multiple imputation dataset. (PDF)

S2 Table Additional analyses of the associations of pre-endoscopy anxiety with study outcomes by limiting the participants among those with complete covariate information. (PDF)

S3 Table Subgroup analyses of the risk of severe discomfort and poor tolerance by each one score increase pre-endoscopy anxiety. (PDF)

S4 Table Subgroup analyses for the risk of severe panic/fear during endoscopy and unwillingness to undergo unsedated endoscopy by each one score increase in pre-endoscopy anxiety. (PDF)

S5 Table Sensitivity analyses. (PDF)
