# Supplementary material for: Associations of anxiety with discomfort and tolerance in Chinese patients undergoing esophagogastroduodenoscopy
Source: PLoS One. 2019 Feb 19;14(2):e0212180. doi: 10.1371/journal.pone.0212180 (PMC6380562; doi:10.1371/journal.pone.0212180)
Supplement: S1 Table — (PDF) [file pone.0212180.s001.pdf]

Supporting information

S1 Table. Additional analyses of the associations of pre-endoscopy anxiety with study outcomes using multiple imputation dataset.

	OR [95%CI]		
	**Unadjusted model**	**Basic model** †	**Fully adjusted model** ‡
**Discomfort**			
OR for 1-score increase in pre-endoscopy anxiety VAS	1.40[1.21, 1.63]	1.43[1.22, 1.69]	1.32[1.09, 1.61]
OR by pre-endoscopy anxiety categories			
Low (0 ≤ VAS ≤ 3)	1.00(referent)	1.00(referent)	1.00(referent)
Moderate (4 ≤ VAS ≤ 6)	3.21[1.54, 6.69]	3.26[1.52, 6.96]	2.70[1.17, 6.22]
High (7 ≤ VAS ≤ 10)	8.15[3.32, 20.03]	9.02[3.52, 23.08]	6.87[2.16, 21.79]
P-trend	<0.001	<0.001	0.001
**Tolerance**			
OR for 1-score increase in pre-endoscopy anxiety VAS	1.60[1.35, 1.90]	1.69[1.39, 2.06]	1.67[1.33, 2.08]*
OR by pre-endoscopy anxiety categories			
Low (0 ≤ VAS ≤ 3)	1.00(referent)	1.00(referent)	1.00(referent)
Moderate (4 ≤ VAS ≤ 6)	4.95[1.92, 12.72]	4.97[1.88, 13.17]	4.58[1.53, 13.70]
High (7 ≤ VAS ≤ 10)	21.75[7.52, 62.88]	28.25[9.16, 87.15]	30.78[7.90, 119.85]
P-trend	<0.001	<0.001	<0.001
**Panic and fear during endoscopy**			
OR for 1-score increase in pre-endoscopy anxiety VAS	1.59[1.32, 1.92]	1.59[1.31, 1.92]	1.80[1.39, 2.32]
OR by pre-endoscopy anxiety categories			
Low (0 ≤ VAS ≤ 3)	1.00(referent)	1.00(referent)	1.00(referent)
Moderate (4 ≤ VAS ≤ 6)	13.64[3.11, 59.86]	13.64[3.11, 59.86]	16.59[3.23, 85.17]
High (7 ≤ VAS ≤ 10)	32.73[6.85, 156.44]	32.73[6.85, 156.44]	88.66[12.32, 637.94]
P-trend	<0.001	<0.001	0.001
**Willingness to repeat unsedated endoscopy**			
OR for 1-score increase in pre-endoscopy anxiety VAS	1.15[1.00, 1.31]	1.14[0.99, 1.31]	1.18[0.99, 1.39]
OR by pre-endoscopy anxiety categories			
Low (0 ≤ VAS ≤ 3)	1.00(referent)	1.00(referent)	1.00(referent)
Moderate (4 ≤ VAS ≤ 6)	1.33[0.71, 2.49]	1.27[0.67, 2.42]	1.61[0.76, 3.39]
High (7 ≤ VAS ≤ 10)	1.86[0.78, 4.43]	1.83[0.76, 4.41]	1.70[0.57, 5.04]
P-trend	0.17	0.21	0.23
