# Supplementary material for: Associations of anxiety with discomfort and tolerance in Chinese patients undergoing esophagogastroduodenoscopy
Source: PLoS One. 2019 Feb 19;14(2):e0212180. doi: 10.1371/journal.pone.0212180 (PMC6380562; doi:10.1371/journal.pone.0212180)
Supplement: S2 Table — (PDF) [file pone.0212180.s002.pdf]

Supporting information

S2 Table. Additional analyses of the associations of pre-endoscopy anxiety with study outcomes by limiting the participants among those with complete covariate information.

	OR [95%CI]		
	**Unadjusted model**	**Basic model** †	**Fully adjusted model** ‡
**Discomfort**			
OR for 1-score increase in pre-endoscopy anxiety VAS	1.33[1.16, 1.52]	1.32[1.15, 1.51]	1.24[1.06, 1.45]
OR by pre-endoscopy anxiety categories			
Low (0 ≤ VAS ≤ 3)	1.00(referent)	1.00(referent)	1.00(referent)
Moderate (4 ≤ VAS ≤ 6)	2.30[1.25, 4.22]	2.15[1.16, 3.96]	1.83[0.94, 3.55]
High (7 ≤ VAS ≤ 10)	5.37[2.42, 11.92]	5.12[2.29, 11.43]	3.45[1.31, 9.08]
P-trend	<0.001	<0.001	0.001
**Tolerance**			
OR for 1-score increase in pre-endoscopy anxiety VAS	1.51[1.30, 1.76]	1.52[1.31, 1.77]	1.53[1.27, 1.83]
OR by pre-endoscopy anxiety categories			
Low (0 ≤ VAS ≤ 3)	1.00(referent)	1.00(referent)	1.00(referent)
Moderate (4 ≤ VAS ≤ 6)	3.83[1.79, 8.21]	4.12[1.89, 9.01]	3.90[1.63, 9.31]
High (7 ≤ VAS ≤ 10)	14.16[5.74, 34.96]	14.83[5.89, 37.33]	13.56[4.51, 40.78]
P-trend	<0.001	<0.001	<0.001
**Panic and fear during endoscopy**			
OR for 1-score increase in pre-endoscopy anxiety VAS	1.58[1.33, 1.87]	1.59[1.33, 1.89]	1.63[1.32, 2.00]
OR by pre-endoscopy anxiety categories			
Low (0 ≤ VAS ≤ 3)	1.00(referent)	1.00(referent)	1.00(referent)
Moderate (4 ≤ VAS ≤ 6)	18.37[4.27, 78.92]	17.03[3.95, 73.51]	16.54[3.70, 73.99]
High (7 ≤ VAS ≤ 10)	38.29[8.10, 181.01]	38.64[8.09, 184.54]	39.91[7.34, 216.84]
P-trend	<0.001	<0.001	0.001
**Willingness to repeat unsedated endoscopy**			
OR for 1-score increase in pre-endoscopy anxiety VAS	1.15[1.02, 1.30]	1.14[1.00, 1.29]	1.12[0.97, 1.29]
OR by pre-endoscopy anxiety categories			
Low (0 ≤ VAS ≤ 3)	1.00(referent)	1.00(referent)	1.00(referent)
Moderate (4 ≤ VAS ≤ 6)	1.31[0.75, 2.29]	1.22[0.69, 2.14]	1.33[0.72, 2.46]
High (7 ≤ VAS ≤ 10)	1.91[0.87, 4.19]	1.85[0.84, 4.11]	1.37[0.54, 3.47]
P-trend	0.12	0.87	0.37
