# Supplementary material for: Associations of anxiety with discomfort and tolerance in Chinese patients undergoing esophagogastroduodenoscopy
Source: PLoS One. 2019 Feb 19;14(2):e0212180. doi: 10.1371/journal.pone.0212180 (PMC6380562; doi:10.1371/journal.pone.0212180)
Supplement: S3 Table — (PDF) [file pone.0212180.s003.pdf]

Supporting information

S3 Table. Subgroup analyses of the risk of severe discomfort and poor tolerance by each one score increase pre-endoscopy anxiety

Subgroup	Discomfort		Tolerance	
	OR[95%CI]	P-interaction	OR[95%CI]	P-interaction
**Age**				
< 35 years	1.24 [1.04, 1.46]*	0.19	1.35 [1.12, 1.63]**	0.12
≥ 35 years	1.42 [1.11, 1.82]**		1.78 [1.32, 2.40]***	
**Sex**				
Men	1.40[1.17, 1.68]***	0.12	1.65[1.33, 2.04]***	0.05
Women	1.17[0.92, 1.50]		1.33[0.99, 1.78]	
**Pharyngitis**				
Yes	1.33[1.12, 1.58]**	0.73	1.59[1.30, 1.96]***	0.89
No	1.42[1.08, 1.88]*		1.57[1.16, 2.13]**	
**Duration of endoscopy**				
< 5 minutes	1.46[1.20, 1.77]***	0.24	1.56[1.28, 1.91]***	0.52
≥ 5 minutes	1.23[0.97, 1.57]		1.48[1.12, 1.97]**	
**Diameter of endoscopy**				
9.0-9.2 mm	1.29[1.09, 1.52]**	0.19	1.49[1.24, 1.78]***	0.75
9.8-9.9 mm	1.77[1.15, 2.73]**		1.58[1.09, 2.30]*	

OR: odds ratio; CI: confidence interval.

* 0.01 ≤ P < 0.05, ** 0.001 ≤ P < 0.01, *** P < 0.001
