# Supplementary material for: Associations of anxiety with discomfort and tolerance in Chinese patients undergoing esophagogastroduodenoscopy
Source: PLoS One. 2019 Feb 19;14(2):e0212180. doi: 10.1371/journal.pone.0212180 (PMC6380562; doi:10.1371/journal.pone.0212180)
Supplement: S4 Table — (PDF) [file pone.0212180.s004.pdf]

Supporting information

S4 Table. Subgroup analyses for the risk of severe panic/fear during endoscopy and unwillingness to undergo unsedated endoscopy by each one score increase in pre-endoscopy anxiety

**Subgroup**	**Panic and fear during endoscopy**		**Willingness to undergo unsedated endoscopy**	
	**OR[95%CI]**	**P-interaction**	**OR[95%CI]**	**P-interaction**
**Age**				
< 35 years	1.43 [1.14, 1.81]**	0.25	1.05 [0.88, 1.24]	0.15
≥ 35 years	1.83 [1.34, 2.50]***		1.26 [1.01, 1.57]*	
**Sex**				
Men	1.62[1.28, 2.06]***	0.70	1.02[0.87, 1.21]	0.13
Women	1.57[1.18, 2.10]**		1.32[1.03, 1.70]***	
**Pharyngitis**				
Yes	1.78[1.40, 2.26]***	0.23	1.11[0.94, 1.31]	0.83
No	1.37[0.99, 1.89]		1.15[0.88, 1.49]	
**Duration of endoscopy**				
< 5 minutes	1.57[1.26, 1.95]***	0.99	1.02[0.86, 1.21]	0.05
≥ 5 minutes	1.53[1.09, 2.13]*		1.40[1.08, 1.81]***	
**Diameter of endoscopy**				
9.0-9.2 mm	1.49[1.22, 1.82]***	0.28	0.94[0.78, 1.12]	0.006
9.8-9.9 mm	2.31[1.21, 4.44]*		1.68[1.18, 2.39]**	

OR: odds ratio; CI: confidence interval.

[*] 0.01 ≤ P < 0.05, [**] 0.001 ≤ P < 0.01, [***] P < 0.001
