# Supplementary material for: Associations of anxiety with discomfort and tolerance in Chinese patients undergoing esophagogastroduodenoscopy
Source: PLoS One. 2019 Feb 19;14(2):e0212180. doi: 10.1371/journal.pone.0212180 (PMC6380562; doi:10.1371/journal.pone.0212180)
Supplement: S5 Table — (PDF) [file pone.0212180.s005.pdf]

Supporting information

S5 Table. Sensitivity analyses

	OR [95%CI] for 1-score increase in pre-endoscopy anxiety VAS			
	**Discomfort**	**Tolerance**	**Panic during endoscopy**	**Willingness to undergo unsedated endoscopy**
Additionally adjust for family income	1.40[1.12, 1.74]	1.89[1.46, 2.45]	2.00[1.47, 2.73]	1.12[0.92, 1.36]
Additionally adjust for snore	1.32[1.09, 1.60]	1.68[1.34, 2.11]	1.86[1.43, 2.42]	1.19[1.00, 1.43]
Additionally adjust for endoscopist	1.34[1.08, 1.65]	1.69[1.32, 2.16]	1.99[1.49, 2.66]	1.15[0.95, 1.40]
Considering VAS ≥ 5 as the definition of high discomfort, poor tolerance, and high panic	1.25[1.07, 1.46]	1.27[1.11, 1.47]	2.01[1.62, 2.49]	1.18[0.99, 1.39]

OR: odds ratio; CI: confidence interval; VAS: visual analogue scale (0 – 10 points).
